# A Multi-Camera System for Bioluminescence Tomography in Preclinical Oncology Research

**DOI:** 10.3390/diagnostics3030325

**Published:** 2013-07-09

**Authors:** Matthew A. Lewis, Edmond Richer, Nikolai V. Slavine, Vikram D. Kodibagkar, Todd C. Soesbe, Peter P. Antich, Ralph P. Mason

**Affiliations:** 1 Department of Radiology, UT Southwestern Medical Center, 5323 Harry Hines Blvd., Dallas, TX 75390, USA; E-Mails: Matthew.Lewis@UTSouthwestern.edu (M.A.L.); richer@lyle.smu.edu (E.R.); Nikolai.Slavine@UTSouthwestern.edu (N.V.S.); Vikram.Kodibagkar@asu.edu (V.D.K.); Todd.Soesbe@UTSouthwestern.edu (T.C.S.); antichino@earthlink.net (P.P.A.); 2 Department of Mechanical Engineering, Southern Methodist University, Dallas, TX 75275, USA; 3 School of Biological and Health Systems Engineering, Arizona State University, Tempe, AZ 85287, USA; 4 Advanced Imaging Research Center, UT Southwestern, Dallas, TX 75390, USA

**Keywords:** imaging systems, medical and biological imaging, tomography, bioluminescence, MRI, lung cancer

## Abstract

Bioluminescent imaging (BLI) of cells expressing luciferase is a valuable noninvasive technique for investigating molecular events and tumor dynamics in the living animal. Current usage is often limited to planar imaging, but tomographic imaging can enhance the usefulness of this technique in quantitative biomedical studies by allowing accurate determination of tumor size and attribution of the emitted light to a specific organ or tissue. Bioluminescence tomography based on a single camera with source rotation or mirrors to provide additional views has previously been reported. We report here *in vivo* studies using a novel approach with multiple rotating cameras that, when combined with image reconstruction software, provides the desired representation of point source metastases and other small lesions. Comparison with MRI validated the ability to detect lung tumor colonization in mouse lung.

## 1. Introduction

The ability of bioluminescence imaging (BLI) to assess gene expression and protein–protein interactions in living animals provides new insight into developmental biology and gene therapy of many diseases [1,2,3,4]. Published investigations report on studies ranging from cardiovascular development to tumor growth and the assessment of diverse promoter elements. The technique is primarily based on the luciferase gene (obtained from the North American firefly, *Photinus pyralis*), which is readily transfected into mammalian cells and expressed effectively [1,4]. The repertoire of transgenes and enzymes together with light emitting substrates is rapidly expanding to include various combinations such as firefly luciferase + *D*-luciferin, renilla luciferase + coelentrazine, Nanoluc + furimazine or β-galactosidase + GalactonPlus [5,6,7,8]. When the appropriate substrate is administered to such cells or to an animal host, a luminescent reaction occurs, emitting light that can be detected and imaged using a cooled charged-coupled device (CCD) digital camera.

BLI is widely used as a planar technique for visualization and localization of cell populations, as well as quantitation tasks such as tumor size estimation. However, the utility of BLI is limited by the fact that the intensity and distribution of the signal captured by the BLI camera at the animal’s skin are strongly influenced by the intervening tissues through both scattering and absorption processes [9,10] and result in an attenuated and diffuse signal at the surface. Bioluminescence tomography (BLT) has been the goal of several research groups, and *in vivo* application has proven to be a relevant method [3,11,12,13,14,15]. The experimental benefits of non-ionization and specificity of targeting in BLT are balanced by an extremely challenging image reconstruction problem. By contrast, a fluorescence molecular tomography system was reported some time ago [16,17]. This method uses external excitation sources, and in fact has an easier inverse problem formulation. There has been a report on imaging of *in vivo* bioluminescence from multiple directions using a rotating mirror system [18], and a commercial system based on a single CCD camera [19] (Xenogen IVIS 3D). Use of a truncated conical mirror was shown to enhance three dimensional (3D) multispectral fluorescence optical tomography in a small animal imaging system [20] and this has since been combined with simultaneous PET acquisition [21]. Gu *et al.* reported three-dimensional bioluminescence tomography with a model based Finite Element algorithm (FEM) [22]. Recently, hyperspectral and multispectral bioluminescent tomography were shown [19,23,24,25,26,27,28,29,30,31], based on imaging with filters at different wavelengths. Multispectral data acquisition solves the problem of underdeterminancy due to a data-object dimensionality mismatch, but presents an optical sensitivity challenge when using narrow filters. Other groups have proposed modulating the boundary conditions [32], temperature [33], or tissue absorption [34] to improve image reconstruction by reducing the ill-posedness of this inverse source problem. Computer simulations for a combined optical-PET system were also shown by Alexandrakis *et al.* [35,36]. In some cases, *a priori* information on the heterogeneous tissue background [37,38] or the nature of the sources [39,40,41,42,43,44] themselves has been incorporated into the image reconstruction strategy. We previously reported an iterative method based on the diffusion equation to reconstruct light emitting sources in phantoms [45] using algorithms from the expectation maximization (EM) family. We now describe a multi camera system for small animal BLI and demonstrate the ability to generate 3D tomographic images of phantoms and tumors growing in mice.

## 2. Experimental Section

### 2.1. Device and Technological Rationale

**Figure S1:**
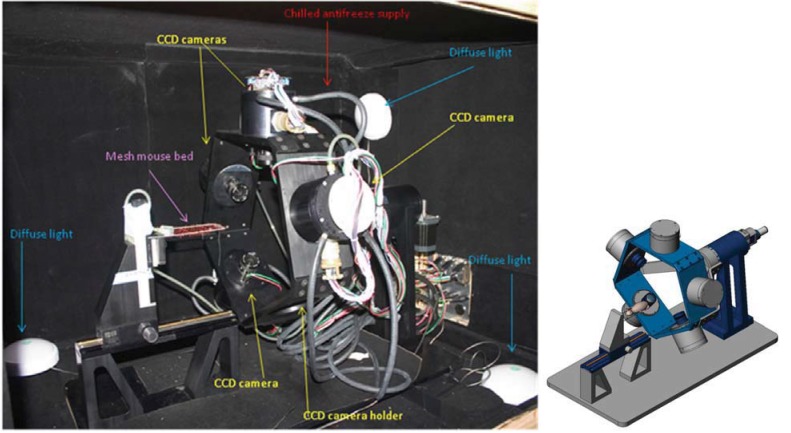
**Left**: view of mBLT apparatus inside light tight box. **Right**: conceptual design drawing for mBLT device.

A significant problem for imaging a tumor or organ within an animal is that intervening tissues can totally or partially obscure the light emitted in the direction of a camera. The light emission may, however, be visible from other viewing angles. Furthermore, bioluminescence is a dynamic process, and light intensity varies significantly and rapidly after substrate administration [46], limiting the amount of time available for imaging. To overcome these problems, it is important to observe as much of the animal surface as possible simultaneously. We therefore designed and built an optical imaging system with multiple cameras that surround the subject in the transverse plane permitting the simultaneous acquisition of images from different angular directions. For this work, we employed four cameras and twenty orientations provided by a computerized system, which rotates the cameras around the longitudinal axis in defined angular steps. An initial five camera conceptual design together with a close up of apparatus are shown in Figure S1.

It is quite evident that the kinetics of luciferin delivery may differ between organs and tissues. In practice, a standard processing technique in BLT has been to remove the temporal component by renormalizing all views based on pre- and post-image from the same direction. Recognizing that complete temporal dynamics could be measured with multiple cameras, the multi-camera bioluminescence tomography system (mBLT) was designed to support quantitative studies of kinetics under the hypothesis that some information about tumor vasculature and perfusion may be inherent to the BLI signal. It has been noted in the literature that the time dependence of BLI sources can perturb the BLT reconstruction [47]. On the other hand, we also note recent literature where the time dependence of *in vivo* optical signals can provide additional information as to the tissue of origin for optical photons [48]. This *a priori* information could also conceivably be used to further constrain and regularize the BLT inverse source problem [49].

The system does not contain filters for performing multispectral imaging. While it is recognized that the addition of spectral information improves the ill-posedness of the BLT image reconstruction problem, parameters for point sources or sources of known configuration can be estimated using either non-linear curve fitting (e.g., Levenberg-Marquardt) or with statistical reconstruction methods. For point sources simulating distant metastatic lesions, we have had good success with an algorithm in the Maximum-Likelihood Expectation-Maximization family [45]. For larger light sources, such as primary tumors (specifically, with spatial dimensions greater than mean free path of photons), the question of superiority of method (planar BLI *vs*. BLT) in determining tumor burden or size is an open issue.

**Figure 1:**
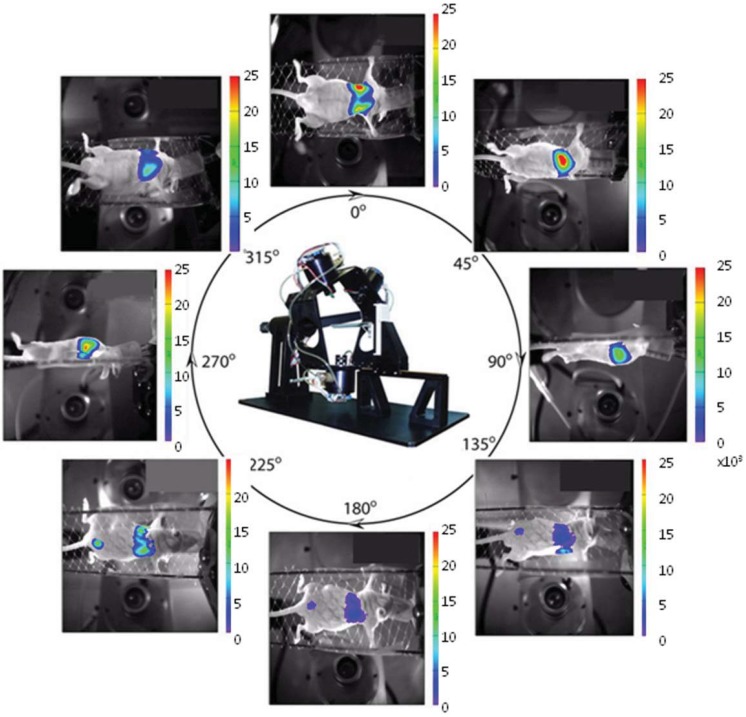
Multi detector bioluminescence imaging (BLI). Eight planar images selected from the twenty obtained with the multi-camera bioluminescence tomography (mBLT) system showing bioluminescent signal overlaid on bright-field images of a nude mouse implanted with A549-luc human tumor cells. Each image was acquired in 60 s following administration of 450 mg/kg *D*-luciferin subcutaneously in the back fore-neck region and is presented with arbitrary relative light units in detector coordinates, so opposite images are inverted.

Figure 1 shows the system and eight representative images showing bioluminescent signal overlaid on external light images. The system uses innovative analysis and reconstruction software capable of providing full 3-D reconstruction and tomographic imaging for bioluminescent tumors that are effectively point sources or small spheres. 

### 2.2. High Sensitivity CCD Camera

The imaging system uses CCD (charge-coupled device) cameras selected for their high and nearly constant sensitivity over the full range of wavelengths commonly used in optical imaging, from blue to near infrared. The CCD (SITe SI-032AB) is a non-color, back-illuminated, full frame image sensor with 512 × 512 pixels, (Scientific Imaging Technologies Inc., Tigard, OR, USA). The quantum efficiency of the CCD is greater than 85% from 400 to 750 nm, and remains above 50% up to 900 nm. The CCD has a pixel size of 24 × 24 μm providing a large well capacity of 350,000 e^−^, with a sensitivity of 2.6 µV/e^−^, low dark current (20 pA/cm^2^ at 20 °C), and low readout noise (5 e^−^ RMS) providing a dynamic range of 75,000. The CCD is cooled to −50 °C reducing the dark current signal to <0.1 e^−^/pixel/s. The large dynamic range of the detector is coupled with a 16-bit analog to digital (A/D) converter, allowing quantitative detection of both high and very low signals, simultaneously, e.g., large tumors and nearby small metastases, or the proximal and distal views of the same tumor from opposite sides of the animal. This is a pivotal property for tomographic data acquisition, as the image intensity can vary by orders of magnitude as a function of the viewing angle. The CCD is incorporated in a self-contained, cooled camera, equipped with electronic circuitry and large aperture optics (25 mm focal length, f/0.95; Figure S1). Each camera is calibrated using a low-intensity, diffuse, flat field source that can be adjusted to a known radiance (typically 3.0 × 10^−7^ W/cm^2^/sr). The light source is periodically checked for uniformity using a NIST-traceable research radiometer (IL 1700, International Light, Inc. Newburyport, MA, USA). By imaging this source the digital units provided by the camera digitizer can be converted directly into absolute physical units (W/cm^2^/sr or photons/s/cm^2^/sr). Moreover, this method accounts for the transmission efficiency of the entire optical system, and corrects the field-of-view non-uniformity due to lens vignetting and variations in pixel sensitivity.

Camera sensitivity depends on several factors including object to image minimization factor (field of view dimensions for a given area of the CCD), f-stop, internal pixel binning, background signal, CCD temperature, and readout noise. Better sensitivity can be achieved by internally binning the signal from the adjacent pixels at the expense of resolution (2 × 2, 4 × 4, and 8 × 8 binning modes are software selectable). Images were processed offline using a Pentium 4 (32-bit, 2 GB RAM) running Linux.

### 2.3. Multiple Head Optical Imaging System

Four high sensitivity CCD cameras simultaneously record views, and a computer controlled rotation mechanism allows imaging at multiple angular positions, required for three dimensional reconstructions. The support electronics allow simultaneous control of the cameras for light exposure, image readout and preprocessing, and temperature and vacuum control. Since each camera in the system is calibrated in absolute units, all resulting images can be directly combined by the reconstruction algorithm. A horizontal bed, made of sparse mesh material to reduce interference with emitted light, is used to support the animal during imaging. A gas anesthesia unit is connected to the bed and to the animal. To exclude ambient light, the system is encapsulated in a light-tight enclosure. Light images at each angular position of the gantry are acquired for co-registration with the bioluminescence image using a set of 6 diffuse light sources that provide uniform illumination of the animal. For the longitudinal tumor growth study in lungs, the imaging time varied from 300 s/angular position for the first imaging sessions that were characterized by low bioluminescent signal, to 30 s/angular position for the later sessions when the bioluminescent signal was large. The average light output of the MDA-MB-231-Luc cells, as measured *in vitro*, was 58 ± 8 photons/s/cell, significantly lower than the 198 ± 14 photons/s/cell measured for the A549-Luc cells. Rotation time between viewing angles was a matter of seconds.

### 2.4. Image Reconstruction Software

Optical imaging methods are subject to the complexity of light transport and practical 3D image reconstruction algorithms for bioluminescence or continuous-wave source imaging have been lacking. Some methods for depth determination from a single view have been reported [50] using multispectral data, and this mode of imaging is now routine in commercial BLI systems.

**Figure S2:**
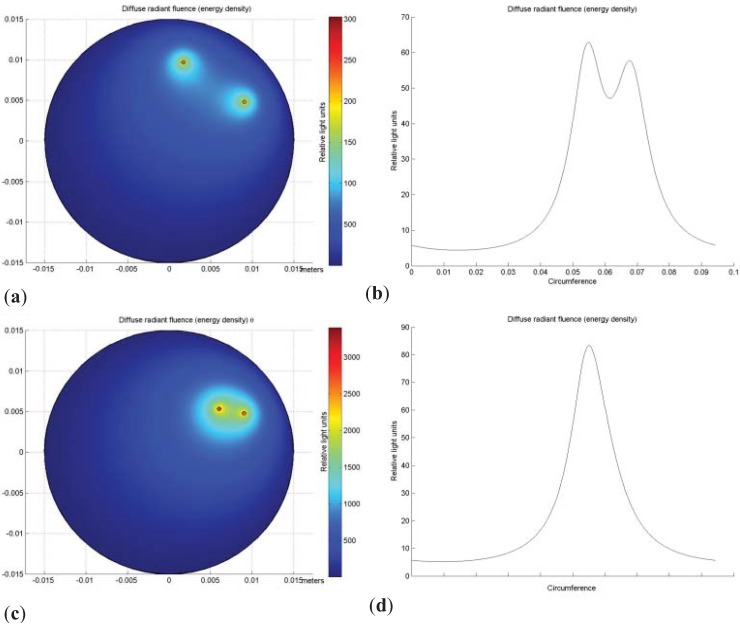
Finite element simulation of surface light distribution for point sources at depth. (**a**) Two point sources modeled using diffusion approximation to RTE, (**b**) sufficiently separated point sources are apparent on surface, (**c**) two neighboring point sources, (**d**) indistinguishable from single, stronger source.

Light that scatters many times during propagation has been modeled extensively using the diffusion approximation to the Boltzmann transport equation. Most theoretical analyses of the bioluminescence tomography inverse problem have adopted this model [51,52,53,54,55]. Application of the diffusion approximation has been described for planar *in vivo* imaging with light-emitting probes [9], but algorithms for light emission tomography have lagged behind developments for optical transmission tomography [16]. In most cases, significant *a priori* information on tissue optical heterogeneity must be included in the image reconstruction procedure [56]. Since attenuation is wavelength dependent, some methods use spectral decomposition to further constrain the reconstruction [23,24,27,56]. The Finite Element Method (FEM) has been used by several groups as the forward solver for iterative, model-based reconstruction [22,57,58], but Monte Carlo methods can also be used [59,60]. As shown in a finite element simulation (COMSOL Multiphysics), even simple imaging scenarios can be problematic in BLT (Figure S2). With 2-point sources in an otherwise homogeneous medium with no detector noise, it may be possible to estimate the number and location of the sources from the light on the surface of the object, provided that they are well-separated. It is easy however to configure 2 sources, so that the distribution of light on the surface is indistinguishable from a single, stronger source (Figure S2).

We previously developed a novel 3D reconstruction algorithm, which could constitute a good base for localizing small sources in small animal imaging in the multi-scattering regime. The reconstruction approach consists of three main steps: external illumination surface reconstruction to determine geometry of turbid medium boundary, initial order approximation of the photon fluence, and an iterative deblurring algorithm for the photon fluence to obtain a final result. A brief description of the algorithm for source reconstruction inside turbid media follows (see Slavine *et al.* [45] and references therein for details):

(1) Estimate the source location by backprojecting the experimental data into the volume discretized into voxels, calculate the intensity of reflection on boundary for each voxel *j*, and subtract its contribution from the transmitted part of intensity. The diffusion equation is used to determine the balance intensities for internal sources *N_S_* for the surface element in a single voxel.
(2) For each voxel *j* determine an initial order approximation for the photon fluence:

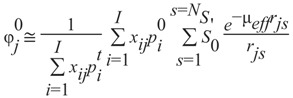
(1)
where *µ_eff_ = (µ_𝑎_/D)^1/2^* The initial photon fluence *φ^o^_j_* depends upon the pathlength from source in voxel *i* to voxel *j* on the surface, intensities in the detectors from source in medium *p^t^_i_*, intensity estimates *p^o^_i_*, the steady-state source power *S^′^_o_*, and the optical properties of the medium *D* and *µ_𝑎_*, the diffusion and absorption coefficients respectively.
(3) Apply the iterative deblurring Expectation Maximization method to obtain a final reconstruction result *φ_j_^n+1^*:

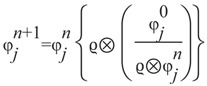
(2)
where ⊗ denotes convolution procedure, ϱ is a Gaussian deblurring kernel, and *n* denotes the iteration number. In practice, the image reconstruction procedure takes 15–20 min on a desktop Linux workstation.

## 3. Results and Discussion

### 3.1. Validation Experiments in a Homogeneous Cylindrical Phantom

**Figure 2:**
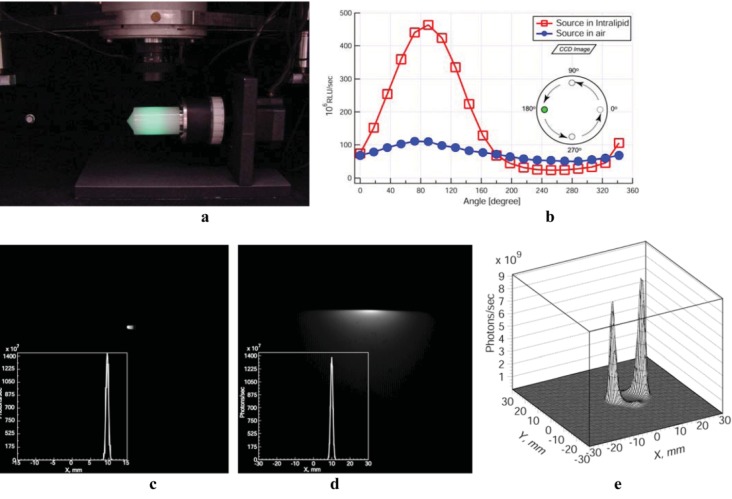
Validation of mBLT in phantoms. (**a**) Experimental setup: 30 mm cylindrical phantom filled with 1% Intralipid gel. (**b**) Total amount of light captured from a fiber optic source in air and in the Intralipid phantom at various angles. The light source is displaced 10 mm from the axis of rotation. Images and intensity profiles through the reconstructed 3D image of light emitting fiber in air (**c**), and Intra-lipid medium (**d**) displaced 10 mm from the center. (**e**) 3D intensity profiles for 1% Intralipid phantom with two point sources separated by 10 mm.

Validation studies in phantoms verified the utility of the approach and accuracy of the tomographic reconstruction algorithms. First tests used a homogeneous diffusing cylinder phantom. The experimental setup consisted of a single camera and a rotating 30 mm diameter cylindrical phantom filled with 1% Intralipid/1% agarose gel (Intralipid gel for brevity), which approximates the light scattering in tissues (Figure 2(a)). A 1 mm diameter optical fiber was placed in the phantom before congealing the filling mixture and was optically coupled to a 560 nm LED, a wavelength appropriate for simulating BLI emission. A set of 20 images was obtained for each experiment by rotating the phantom 18° at a time. The mBLT system is not currently optimized for number of views. While it may be possible to image the surface intensity with fewer views while maintaining SNR, the importance of surface obliqueness factors (determined by surface normals) is increased. We note that the trade-off between image quality, the number of non-contact optical projections, and exposure time for constant sources in turbid media is an important consideration for enhancing BLT for dynamic sources. The total amount of light obtained with a constant intensity light source and captured at each imaging angle either in air or the Intralipid phantom is presented in Figure 2(b). The light source in air showed only a small variation in the recorded signal due to changes in distance from the lens. When the source was immersed in the Intralipid gel it was noted that for angles between 0° and 180°, when the light source was closest to the camera, up to 4 times more light was captured than for the source in air, as the scattering of light in the phantom caused a significant increase in the photon flux toward the lens. When the source was in the position furthest from the camera (270°) only half as much light was captured, because the light source was shadowed by the body of the phantom. Overall, a variation of almost an order of magnitude was observed when imaging this phantom at different angles, showing the limited precision and reliability of a quantitative assessment from single planar images, even with minimal absorption from chromophores. This effect may be expected to affect animal studies, when light is emitted at depth within the body from multiple sources, such as small metastatic tumors in the lungs or other internal organs.

Figure 2(c, d) show images obtained in air and in Intralipid gel respectively, with the fiber displaced 10 mm from the center at a viewing angle of 180°. A cross-section through the reconstructed volumetric image is also shown for each case. The position of the source was correctly identified by the reconstruction algorithm. The total reconstructed signal in air and Intralipid differed by only 3% (1.435 × 10^10^ photons/s for air *vs*. 1.390 × 10^10^ photons/s for Intralipid), further showing the quantitative nature of the volumetric reconstruction algorithm. Confirmatory results were also obtained for multiple light sources, both in air and Intralipid, e.g., for two sources separated by 10 mm (Figure 2(e)).

### 3.2. In Vivo Imaging in Lung Metastasis Models

*In vivo* imaging was tested in a model of progressing lung metastases with direct comparison to MRI. Human lung tumor cells (10^6^ A549-luc cells stably transfected with the luciferase gene) were injected in the tail vein of a nude mouse (BALBc/nu/nu), as described previously [61]. The average light output of 198 ± 14 photons/s/cell was measured *in vitro* by the method of Troy *et al.* [62]. After 63 days the lung-colonizing experimental metastases were imaged following *D*-luciferin injection (450 mg/kg, SQ) in the anesthetized mouse [63]. A set of 20 images, 18° apart, was obtained using 1 min exposure starting 3 min post-injection of *D*-luciferin (Figure 1). An externally illuminated image of the mouse was obtained for co-registration at each camera position. 

**Figure 3:**
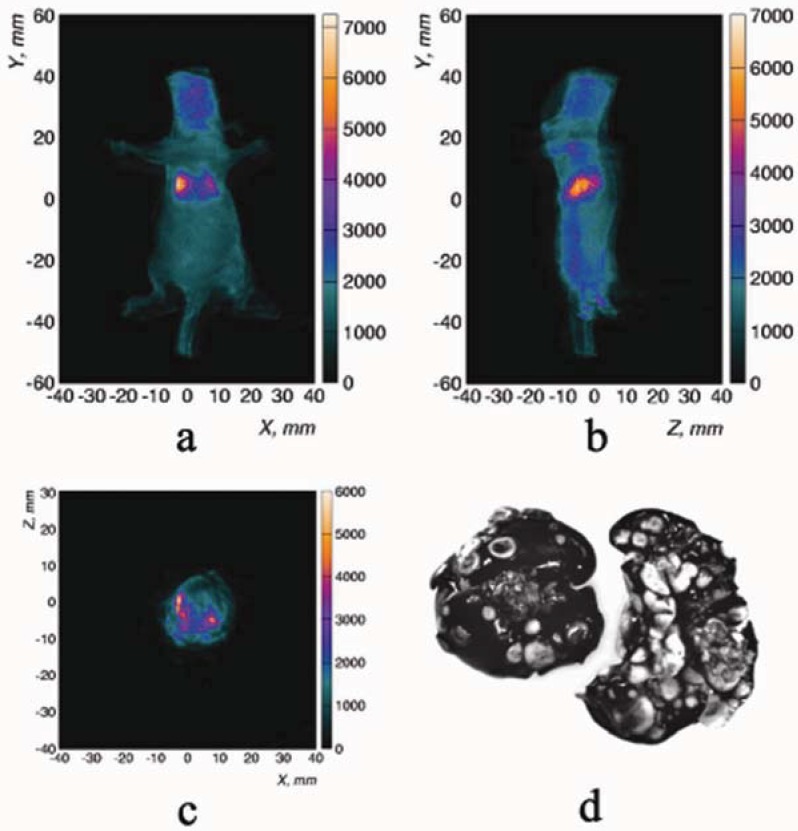
Projections from 3D reconstruction of tumor bearing mouse. (**a**) Coronal and (**b**) sagittal maximum intensity projections, and (**c**) transverse section of 3D bioluminescence tomography (BLT) reconstruction showing A549-luc tumors in mouse lungs (0.5 mm voxel dimensions). (**d**) Staining of intact lungs by injection of India ink directly into the trachea indicates approximately 10^3^ foci. BLT images are fused with external illumination surface reconstructions.

**Figure S3:**
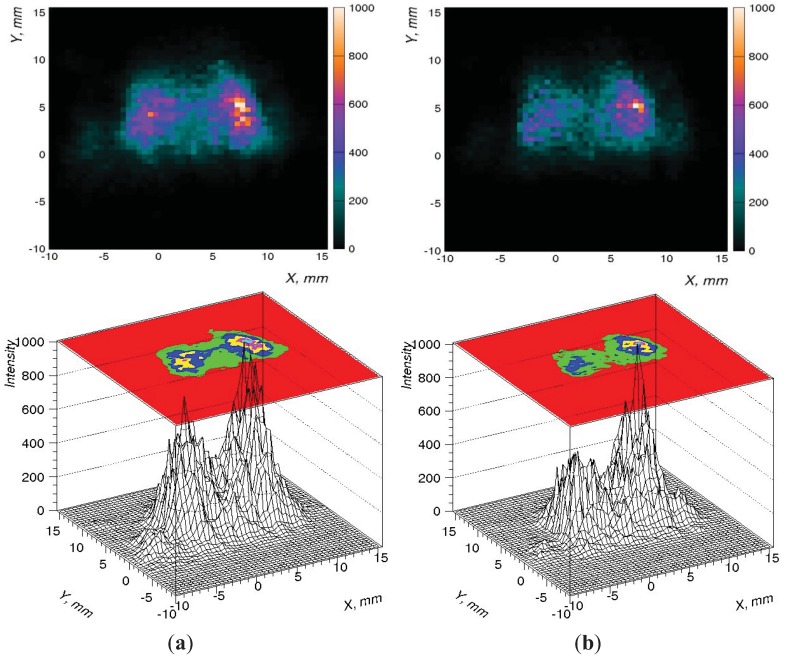
Correlation of the surface radiance (**top**) and voxel populations (**bottom**) with number of projections included in image reconstruction procedure after 10 iterations with voxel size 0.5 mm^3^ (grid 160 × 240 × 160). (**a**) Reconstruction from 20 views, (**b**) Reconstruction from 10 views. The surface radiance is quite similar to the original photograph captured at 0° in Figure 1.

Data reconstruction used the approach described above [45] and semi-automated image processing [64] to provide a 3-D model of the lung tumors (Figure 3). Predicted surface radiance based on the voxel populations in the 3D reconstruction is shown in Figure S3. We also performed a reconstruction using only half the angular views and found quite similar pattern, though with lower signal to noise (Figure S3(b)). Intense local foci are apparent with a more diffuse background distribution outlining the anatomy of the lungs. Pathologic studies after sacrifice confirmed the presence of A549-luc tumor cells dispersed throughout the lobes of the lungs with multiple metastatic foci. Staining of intact lungs by injection of India ink directly into the trachea indicated approximately 10^3^ metastatic foci per mouse (Figure 3(d)), as confirmed by sectioning of histological specimens (data not shown). 

### 3.3. Multimodality in Vivo Comparison

**Figure S4:**
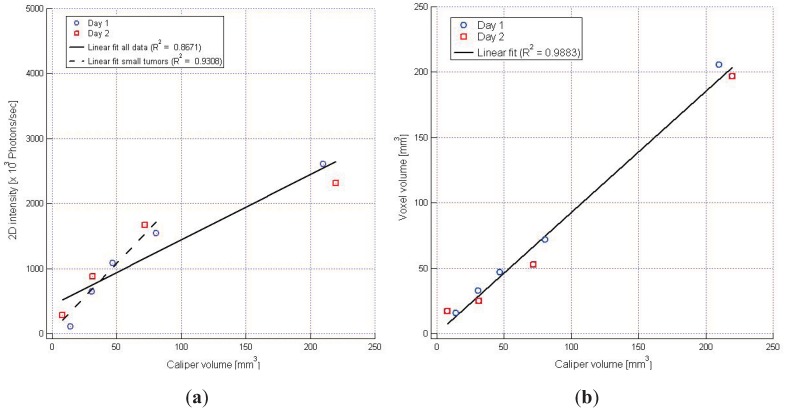
Correlation of caliper measurements *vs*. (i) planar bioluminescence and (ii) mBLT reconstructed volume. PC3-Luc cells injected in the flank of nude mice (BALBc/nu/nu) and imaged in the mBLT system using 5 s exposure time from 20 angular directions (4 cameras × 5 positions) on successive occasions over a period of weeks. (**a**) The 0° angular view was used as a representative planar image and the total light emission was calculated and compared with the volume calculated from caliper measurements of the tumors. (**b**) Volumetric images of the tumors were reconstructed using the BLT algorithm and 0.5 mm cubic voxels. Each tumor volume was estimated using the volume of the voxels exhibiting bioluminescence intensity above the background and compared with the volume obtained from caliper measurements. For small tumors the planar BLI yields good correlation with the caliper measurements (**a**), but when the larger tumors are considered the correlation became weaker (R^2^ = 0.93 *vs.* R^2^ = 0.86). The volume calculated using the volumetric BLT algorithm exhibits better correlation with the caliper measurements (R^2^ = 0.988) and there was no degradation of the linearity for large tumors. Moreover, the linear regression has a zero intercept and a slope close to unity (0.925) indicating equivalence of the two measurements. On 4 occasions the BLI was repeated on consecutive days indicating reproducibility of observations.

As a first validation we tested human PC3-luc prostate tumor cells growing subcutaneously in the flank of a nude mouse. Repeated measurements over a period of weeks confirmed a close correlation between planar BLI signal intensity and caliper measured tumor volume when tumors were small (Figure S4), as expected [46,65]. However, for larger tumor volume the linearity appeared less robust, which is generally attributed to light self-absorption by the tumor and has been reported previously [66]. For the 3D reconstruction, excellent linearity was observed up to the maximum tumor size tested, about 200 mm^3^ (Figure S4(b)). 

**Figure 4:**
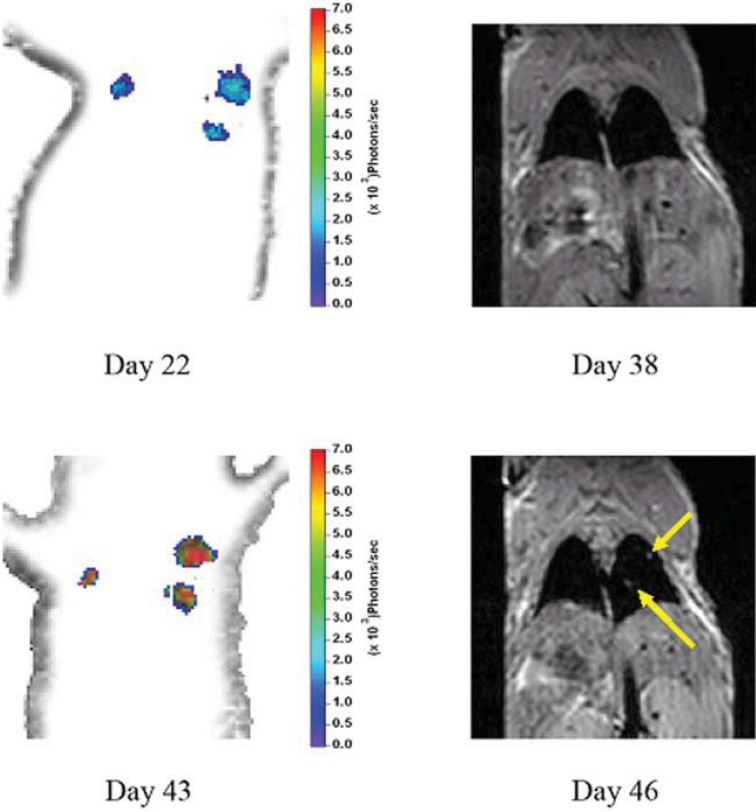
Comparison of mBLT and MRI taken at different time points in MDA-MB-231-luc lung metastasis model. Coronal slices are 1 mm thick, and they correspond to the same position in the animal. mBLT was able to localize the tumors and detect them 17 days earlier. MRI did not detect the smaller tumor in the left lung. Contiguous spin-echo proton density weighted MR coronal slices were obtained with a 4.7 T Varian scanner.

**Figure 5:**
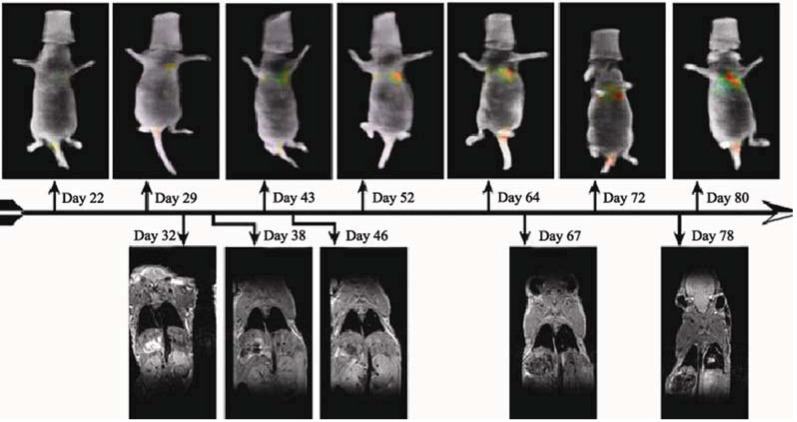
Longitudinal imaging of lung tumor bearing nude mouse. After lung tumors became MRI-detectable on day 46, increasing tumor burden observed using repeated BLT was confirmed by MRI. MRI did not detect the smaller tumor in the left lung. The BLT imaging time varied from 300 s/angular position for the first imaging sessions that were characterized by low bioluminescent signal, to 30 s/angular position for the later sessions when the bioluminescent signal was large.

Subsequent comparison used MRI to examine human breast tumor cells (10^6^ MDA-MB-231-luc) following IV injection in a nude mouse. The animal was imaged, using the same protocol as described above (Section 3.2), at different time points as the lung-colonizing metastases appeared and progressed in size and number. The imaging time varied from 300 s/angular position for the first imaging sessions that were characterized by low bioluminescent signal, to 30 s/angular position for the later sessions when the bioluminescent signal was large. The average light output of these cells, as measured *in vitro*, was 58 ± 8 photons/s/cell, which was significantly lower than for the A549-luc cells. MRI scans covering the chest of the mouse were acquired on a 4.7 T Unity Inova system (Varian Inc., Palo Alto, CA, USA) using a custom built respiratory gating unit [67]. Contiguous spin-echo proton density weighted MR coronal slice images were acquired (TE = 12 ms, FOV = 3.2 cm × 6.4 cm, slice thickness = 1 mm, matrix = 64 × 128 zero filled to 128 × 256, 4 averages). The mBLT images first detected a bioluminescent signal 22 days after cell implantation, and followed the growth and spread of the lung metastases at weekly intervals. Tumors in the right lungs were first detected by MRI 46 days after inoculations (Figure 4). The position and relative sizes of the tumors were consistent between the two imaging modalities. The smaller tumors in the left lung shown on the mBLT images were not observed by MRI on day 46. This is consistent with the fact that the tumors detectable by mBLT on day 22 were not detected in the MR images taken 38 days after implantation. As shown in Figure 5, increasing tumor burden in the lung was observed using serial BLT imaging. This was confirmed by MRI of the growing lesion in the right lung. By comparison, CT images taken with a GE CT/I scanner on days 40 and 47 failed to detect the tumors (data not shown).

All tomographic imaging modalities and their respective reconstruction algorithms have limitations in terms of the required minimum signal intensities, signal to noise ratio, and the number of projections necessary to obtain good quality images. In the case of mBLT, when the bioluminescent signal is very low due to either small tumor volume (incipient metastases), and/or low light production efficiency of the cell line, several angular directions could yield images with no detectable signal due to intervening tissue with high absorption properties. Thus, it will be important to determine the limits of mBLT regarding the smallest signal that produces accurate volumetric images in a realistic experimental setup.

## 4. Conclusions

We have demonstrated tomographic visualization of luciferase expressing cells at depth in a living mouse. The optical imaging system is based on multiple high sensitivity cooled CCD cameras that rotate around the object and on an image reconstruction algorithm providing a three-dimensional reconstruction of the data in the multidirectional planar bioluminescence images. The method can provide both volumetric and tomographic representations, and could potentially provide better quantification of the light emission than simple planar imaging techniques. In the case of deeper tumors in the animal, the quantifying power of the planar BLI technique is expected to degrade significantly. It has also been reported that less light is detected from large tumors than would be expected for their volume potentially due to tissue self-absorption [66]. A tomographic method such as mBLT may be superior to planar BLI in certain instances, but we do note that the added complexity and acquisition times of 3D BLI have hitherto detracted from its widespread application. The use of multiple cameras should accelerate data acquisition compared with a single camera system, but obviously increases costs considerably

The mBLT has the potential to enhance the ability to assess and monitor spatio-temporal characteristics of tumor growth, identify metastases, and potentially determine the effectiveness of cancer treatment. Since the technique is non-invasive, animals may be imaged for the entire course of an investigation, including tumor initiation, growth, treatment, and re-growth significantly reducing the number of animals required for the study. 

## References

[1] Thorne S.H., Contag C.H. (2005). Using *in vivo* bioluminescence imaging to shed light on cancer biology. Proc. IEEE.

[2] Villalobos V., Naik S., Piwnica-Worms D. (2007). Current state of imaging protein-protein interactions *in vivo* with genetically encoded reporters. Annu. Rev. Biomed. Eng..

[3] Weissleder R., Pittet M.J. (2008). Imaging in the era of molecular oncology. Nature.

[4] O’Neill K., Lyons S.K., Gallagher W.M., Curran K.M., Byrne A.T. (2009). Bioluminescent imaging: A critical tool in pre-clinical oncology research. J. Pathol..

[5] Prescher J.A., Contag C.H. (2010). Guided by the light: Visualizing biomolecular processes in living animals with bioluminescence. Curr. Opin. Chem. Biol..

[6] Hall M.P., Unch J., Binkowski B.F., Valley M.P., Butler B.L., Wood M.G., Otto P., Zimmerman K., Vidugiris G., Machleidt T. (2012). Engineered luciferase reporter from a deep sea shrimp utilizing a novel imidazopyrazinone substrate. ACS Chem. Biol..

[7] Liu L., Mason R.P. (2010). Imaging β-galactosidase activity in human tumor xenografts and transgenic mice using a chemiluminescent substrate. PLoS ONE.

[8] Bhaumik S., Lewis X.Z., Gambhir S.S. (2004). Optical imaging of Renilla luciferase, synthetic Renilla luciferase, and firefly luciferase reporter gene expression in living mice. J. Biomed. Opt..

[9] Rice B.W., Cable M.D., Nelson M.B. (2001). *In vivo* imaging of light-emitting probes. J. Biomed. Opt..

[10] Tromberg B.J., Shah N., Lanning R., Cerussi A., Espinoza J., Pham T., Svaasand L., Butler J. (2000). Non-invasive *in vivo* characterization of breast tumors using photon migration spectroscopy. Neoplasia.

[11] Wang G., Cong W., Shen H., Qian X., Henry M., Wang Y. (2008). Overview of bioluminescence tomography—A new molecular imaging modality. Front. Biosci..

[12] Roncali E., Savinaud M., Levrey O., Rogers K.L., Maitrejean S., Tavitian B. (2008). New device for real-time bioluminescence imaging in moving rodents. J. Biomed. Opt..

[13] Yan H., Lin Y., Barber W.C., Unlu M.B., Gulsen G. (2012). A gantry-based tri-modality system for bioluminescence tomography. Rev. Sci. Instrum.

[14] Feng J., Qin C., Jia K., Zhu S., Yang X., Tian J. (2012). Bioluminescence tomography imaging *in vivo*: Recent advances. IEEE J. Sel. Top. Quant. Electron..

[15] Cong W., Wang G., Kumar D., Liu Y., Jiang M., Wang L., Hoffman E., McLennan G., McCray P., Zabner J., Cong A. (2005). Practical reconstruction method for bioluminescence tomography. Opt. Express.

[16] Ntziachristos V., Weissleder R. (2001). Experimental three-dimensional fluorescence reconstruction of diffuse media by use of a normalized born approximation. Optics Letters.

[17] Graves E.E., Ripoll J., Weissleder R., Ntziachristos V. (2003). A submillimeter resolution fluorescence molecular imaging system for small animal imaging. Med. Phys..

[18] Kok P., Botha C.P., Dijkstra J., Kaijzel E., Que I., Löwik C.W.G.M., Reiber J.H.C., Lelieveldt B.P.F., Post F.H. (2007). Integrated visualization of multi-angle bioluminescence imaging and micro CT. Proc. SPIE.

[19] Kuo C., Coquoz O., Troy T.L., Xu H., Rice B.W. (2007). Three-dimensional reconstruction of *in vivo* bioluminescent sources based on multispectral imaging. J. Biomed. Opt..

[20] Li C., Mitchell G.S., Dutta J., Ahn S., Leahy R.M., Cherry S.R. (2009). A three-dimensional multispectral fluorescence optical tomography imaging system for small animals based on a conical mirror design. Opt. Express.

[21] Li C., Yang Y., Mitchell G.S., Cherry S.R. (2011). Simultaneous PET and multispectral 3-dimensional fluorescence optical tomography imaging system. J. Nucl. Med..

[22] Gu X., Zhang Q., Larcom L., Jiang H. (2004). Three-dimensional bioluminescence tomography with model-based reconstruction. Opt. Express.

[23] Chaudhari A.J., Darvas F., Bading J.R., Moats R.A., Conti P.S., Smith D.J., Cherry S.R., Leahy R.M. (2005). Hyperspectral and multispectral bioluminescence optical tomography for small animal imaging. Phys. Med. Biol..

[24] Dehghani H., Davis S.C., Jiang S., Pogue B.W., Paulsen K.D., Patterson M.S. (2006). Spectrally resolved bioluminescence optical tomography. Optics Letters.

[25] Wang G., Shen H., Liu Y., Cong A., Cong W., Wang Y., Dubey P. (2008). Digital spectral separation methods and systems for bioluminescence imaging. Opt. Express.

[26] Dehghani H., Davis S.C., Pogue B.W. (2008). Spectrally resolved bioluminescence tomography using the reciprocity approach. Med. Phys..

[27] Ahn S., Chaudhari A.J., Darvas F., Bouman C.A., Leahy R.M. (2008). Fast iterative image reconstruction methods for fully 3D multispectral bioluminescence tomography. Phys. Med. Biol..

[28] Lu Y., Zhang X., Douraghy A., Stout D., Tian J., Chan T.F., Chatziioannou A.F. (2009). Source reconstruction for spectrally-resolved bioluminescence tomography with sparse a priori information. Opt. Express.

[29] Feng J., Qin C., Jia K., Han D., Liu K., Zhu S., Yang X., Tian J. (2011). An adaptive regularization parameter choice strategy for multispectral bioluminescence tomography. Med. Phys..

[30] Qin C., Yang X., Feng J., Liu K., Liu J., Yan G., Zhu S., Xu M., Tian J. (2009). Adaptive improved element free Galerkin method for quasi- or multi-spectral bioluminescence tomography. Opt. Express.

[31] Virostko J.M., Powers A.C., Jansen E.D. (2008). Validation of luminescent source reconstruction using spectrally resolved bioluminescence images. Proc. SPIE.

[32] Soloviev V.Y. (2007). Tomographic bioluminescence imaging with varying boundary conditions. Appl. Optics.

[33] Wang G., Shen H., Cong W., Zhao S., Wei G.W. (2006). Temperature-modulated bioluminescence tomography. Opt. Express.

[34] Jansen E.D., Pickett P.M., Mackanos M.A., Virostko J. (2006). Effect of optical tissue clearing on spatial resolution and sensitivity of bioluminescence imaging. J. Biomed. Opt..

[35] Alexandrakis G., Rannou F.R., Chatziioannou A.F. (2006). Effect of optical property estimation accuracy on tomographic bioluminescence imaging: Simulation of a combined optical-PET (OPET) system. Phys. Med. Biol..

[36] Alexandrakis G., Rannou F.R., Chatziioannou A.F. (2005). Tomographic bioluminescence imaging by use of a combined optical-PET (OPET) system: A computer simulation feasibility study. Phys. Med. Biol..

[37] Yan H., Unlu M.B., Nalcioglu O., Gulsen G. (2010). Bioluminescence tomography with structural and functional a priori information. Proc. SPIE.

[38] Naser M.A., Patterson M.S., Wong J.W. (2012). Self-calibrated algorithms for diffuse optical tomography and bioluminescence tomography using relative transmission images. Biomed. Optics Express.

[39] Feng J., Qin C., Jia K., Zhu S., Liu K., Han D., Yang X., Gao Q., Tian J. (2012). Total variation regularization for bioluminescence tomography with the split Bregman method. Appl. Optics.

[40] Guo W., Jia K., Zhang Q., Liu X., Feng J., Qin C., Ma X., Yang X., Tian J. (2012). Sparse reconstruction for bioluminescence tomography based on the semigreedy method. Comput. Math. Methods Med..

[41] Guo W., Jia K., Tian J., Han D., Liu X., Wu P., Feng J., Yang X. (2012). An efficient reconstruction method for bioluminescence tomography based on two-step iterative shrinkage approach. Proc. SPIE.

[42] Guo W., Jia K., Tian J., Han D., Liu X., Liu K., Zhang Q., Feng J., Qin C. (2012). Sparsity reconstruction for bioluminescence tomography based on an augmented lagrangian method. Proc. SPIE.

[43] Qin C., Zhu S., Feng J., Zhong J., Ma X., Wu P., Tian J. (2011). Comparison of permissible source region and multispectral data using efficient bioluminescence tomography method. J. Biophoton..

[44] Zhang B., Yang X., Qin C., Liu D., Zhu S., Feng J., Sun L., Liu K., Han D., Ma X. (2010). A trust region method in adaptive finite element framework for bioluminescence tomography. Opt. Express.

[45] Slavine N.V., Lewis M.A., Richer E., Antich P.P. (2006). Iterative reconstruction method for light emitting sources based on the diffusion equation. Med. Phys..

[46] Paroo Z., Bollinger R.A., Braasch D.A., Richer E., Corey D.R., Antich P.P., Mason R.P. (2004). Validating bioluminescence imaging as a high-throughput, quantitative modality for assessing tumor burden. Mol. Imag..

[47] Unlu M.B., Gulsen G. (2008). Effects of the time dependence of a bioluminescent source on the tomographic reconstruction. Appl. Optics.

[48] Hillman E.M.C., Moore A. (2007). All-optical anatomical co-registration for molecular imaging of small animals using dynamic contrast. Nat. Photon..

[49] Feng J., Jia K., Yan G., Zhu S., Qin C., Lv Y., Tian J. (2008). An optimal permissible source region strategy for multispectral bioluminescence tomography. Opt. Express.

[50] Virostko J., Powers A.C., Jansen E.D. (2007). Validation of luminescent source reconstruction using single-view spectrally resolved bioluminescence images. Appl. Optics.

[51] Han W.M., Wang G. (2007). Theoretical and numerical analysis on multispectral bioluminescence tomography. IMA J. Appl. Math..

[52] Han W.M., Cong W.X., Wang G. (2006). Mathematical theory and numerical analysis of bioluminescence tomography. Inverse Probl..

[53] Wang G., Li Y., Jiang M. (2004). Uniqueness theorems in bioluminescence tomography. Med. Phys..

[54] Cheng X.L., Gong R.F., Han W.M. (2008). A new general mathematical framework for bioluminescence tomography. Comput. Meth. Appl. Mech. Eng..

[55] Han W.M., Wang G. (2008). Bioluminescence tomography: Biomedical background, mathematical theory, and numerical approximation. J. Comput. Math..

[56] Lv Y., Tian J., Cong W., Wang G., Yang W., Qin C., Xu M. (2007). Spectrally resolved bioluminescence tomography with adaptive finite element analysis: Methodology and simulation. Phys. Med. Biol..

[57] Lv Y., Tian J., Cong W., Wang G., Luo J., Yang W., Li H. (2006). A multilevel adaptive finite element algorithm for bioluminescence tomography. Opt. Express.

[58] Gong W., Li R., Yan N., Zhao W. (2008). An improved error analysis for finite element approximation of bioluminescence tomography. J. Comput. Math..

[59] Kumar D., Cong W.X., Wang G. (2007). Monte Carlo method for bioluminescence tomography. Indian J. Exp. Biol..

[60] Li H., Tian J., Zhu F., Cong W., Wang L.V., Hoffman E.A., Wang G. (2004). A mouse optical simulation environment (MOSE) to investigate bioluminescent phenomena in the living mouse with the Monte Carlo method. Acad. Radiol..

[61] Dikmen Z.G., Gellert G., Dogan P., Mason R., Antich P., Richer E., Wright W.E., Shay J.E. (2005). A new diagnostic system in cancer research: Bioluminescent imaging (BLI). Turk. J. Med. Sci..

[62] Troy T., Jekic-McMullen D., Sambucetti L., Rice B. (2004). Quantitative comparison of the sensitivity of detection of fluorescent and bioluminescent reporters in animal models. Mol. Imag..

[63] Contero A., Richer E., Gondim A., Mason R.P. (2009). High-throughput quantitative bioluminescence imaging for assessing tumor burden. Methods Mol. Biol..

[64] Slavine N.V., McColl R.W., Richer E., Mason R.P., Antich P.P. An Automated 3D Image-Processing Strategy for Small-Animal Bioluminescence Cancer Studies. Proceedings of Biotechnology and Bioinformatics Symposium (BIOT-2008).

[65] Alhasan M.K., Liu L., Lewis M.A., Magnusson J., Mason R.P. (2012). Comparison of optical and power Doppler ultrasound imaging for non-invasive evaluation of arsenic trioxide as a vascular disrupting agent in tumors. PLoS ONE.

[66] Sarraf-Yazdi S., Mi J., Dewhirst M.W., Clary B.M. (2004). Use of *in vivo* bioluminescence imaging to predict hepatic tumor burden in mice. J. Surg. Res..

[67] Garbow J.R., Zhang Z., You M. (2004). Detection of primary lung tumors in rodents by magnetic resonance imaging. Cancer Res..

